# Inverse design of photonic meta-structure for beam collimation in on-chip sensing

**DOI:** 10.1038/s41598-021-84841-2

**Published:** 2021-03-05

**Authors:** Robin Singh, Yuqi Nie, Mingye Gao, Anuradha Murthy Agarwal, Brian W. Anthony

**Affiliations:** 1 Department of Mechanical Engineering, Massachusetts Institute of Technology, Cambridge, MA 02139 USA; 2 Institute for Medical Engineering and Science, Massachusetts Institute of Technology, Cambridge, MA 02139 USA; 3 Department of Materials Science and Engineering, Massachusetts Institute of Technology, Cambridge, MA 02139 USA; 4 Microphotonics Center, Massachusetts Institute of Technology, Cambridge, MA 02139 USA; 5 Department of Electrical Engineering and Computer Science, Massachusetts Institute of Technology, Cambridge, MA 02139 USA; 6 Materials Research Laboratory, 6 MIT. Nano, Massachusetts Institute of Technology, Cambridge, MA 02139 USA

**Keywords:** Optics and photonics, Applied optics, Lasers, LEDs and light sources, Optical materials and structures, Optical physics, Optical techniques, Other photonics

## Abstract

Designed or patterned structured surfaces, metasurfaces, enable the miniaturization of complex arrangements of optical elements on a plane. Most of the existing literature focuses on miniaturizing the optical detection; little attention is directed to on-chip optical excitation. In this work, we design a metasurface to create a planar integrated photonic source beam collimator for use in on-chip optofluidic sensing applications. We use an iterative inverse design approach in order to optimize the metasurface to achieve a target performance using gradient descent method. We then fabricate beam collimators and experimentally compare performance characteristics with conventional uniform binary grating-based photonic beam diffractors. The optimal design enhances the illumination power by a factor of 5. The reinforced beam is more uniform with 3 dB beam spot increased almost ~ 3 times for the same device footprint area. The design approach will be useful in on-chip applications of fluorescence imaging, Raman, and IR spectroscopy and will enable better multiplexing of light sources for high throughput biosensing.

## Introduction

The promise of silicon photonics is that the technology will enable flexible, low-cost, and scalable approaches for the miniaturization of integrated electronic and photonic systems^1–3^, enabling on-chip spectroscopic sensing and imaging techniques in the fields of medicine and biology^4–6^. Common techniques, and candidates for miniaturization, include fluorescence imaging/microscopy, infra-red (IR) spectroscopy, and Raman spectroscopy^1, 7–12^.

Fluorescence imaging/microscopy is a powerful tool for biomedical research; it provides very high sensitivity and specificity for cellular activity detection, making it the gold standard. Over the past decade, with the advent of semiconductor image sensing technology^13, 14^, researchers have demonstrated on-chip contact-based fluorescence detection techniques with high throughput and scalability^11^. Takeshara et al. implemented contact fluorescence microscopy in microfluidic chips^13^. Pang et al. developed various optofluidic devices that can perform fluorescence detection, imaging and sensing^8^.

Similarly, IR and Raman spectroscopic techniques, based on phononic vibrational states of molecules, are common methods in chemistry. These vibrational spectroscopic techniques are label-free methods, and researchers are interested in miniaturizing them to develop high throughput and high-resolution *lab-on-chip* sensors. Perichetti et al. developed a multifunctional platform for Raman and fluorescence spectroscopic analysis^12^. Chen et al. investigated on-chip methods for Surface Raman spectroscopy integrated with thin layer chromatography^15^. IMEC recently demonstrated SiN waveguide-based on-chip Raman spectroscopy that promises high throughput and better spectral resolution^16^.

Most research attention to date, for miniaturization of these techniques, focuses on developing on-chip detector solutions. Relatively little attention is directed to on-chip excitation solutions with researchers instead using benchtop laser beams or LED-based sources for excitation^8, 13^. In addition to the limit of large instrumentation, a major disadvantage of this method is that photonic excitation performed using LED/benchscale lasers is not spatially confined. This is predominantly due to diffraction limited beam propagation which results in high background noise, leading to poor signal-to-noise (SNR) ratio and sensitivity.

A miniature, on-chip, platform for both excitation and detection will offer several advantages. First, the field-of-view for imaging is easily scaled up by multiplexing a large number of excitation sources. Second, it provides a compact hardware geometry to implement solutions at a low-cost with high volume production. Third, it enables small sample biological analysis, such as cell screening, probing, and automation.

The proposed photonic waveguide-based metasurface design approach will enable the miniaturization of excitation sources, and will include integrated on-chip photon routing and manipulation. Researchers have previously demonstrated waveguides which guide and diffract light, out of the propagation plane, using conventional grating surfaces. For example, Kerman et al. used a conventional focused grating coupler design to excite samples in a microfluidic chip^17^. The dominant focus for other designs has been to enhance fiber coupling to photonic integrated circuits^18–20^. These gratings employ a high degree of symmetry in their structure and fail to create uniform out of-the-plane collimation. In a microfluidic application, with a liquid sample flowing through the light-path in an integrated microfluidic channel, non-uniform excitation results in spatially non-uniform measurements of the fluid. An artificial diffracting (meta) surface that can collimate the beam, uniformly out of the plane over a wide area, will enhance on-chip microfluidic sensing applications^18, 21–23^.

**Figure 1:**
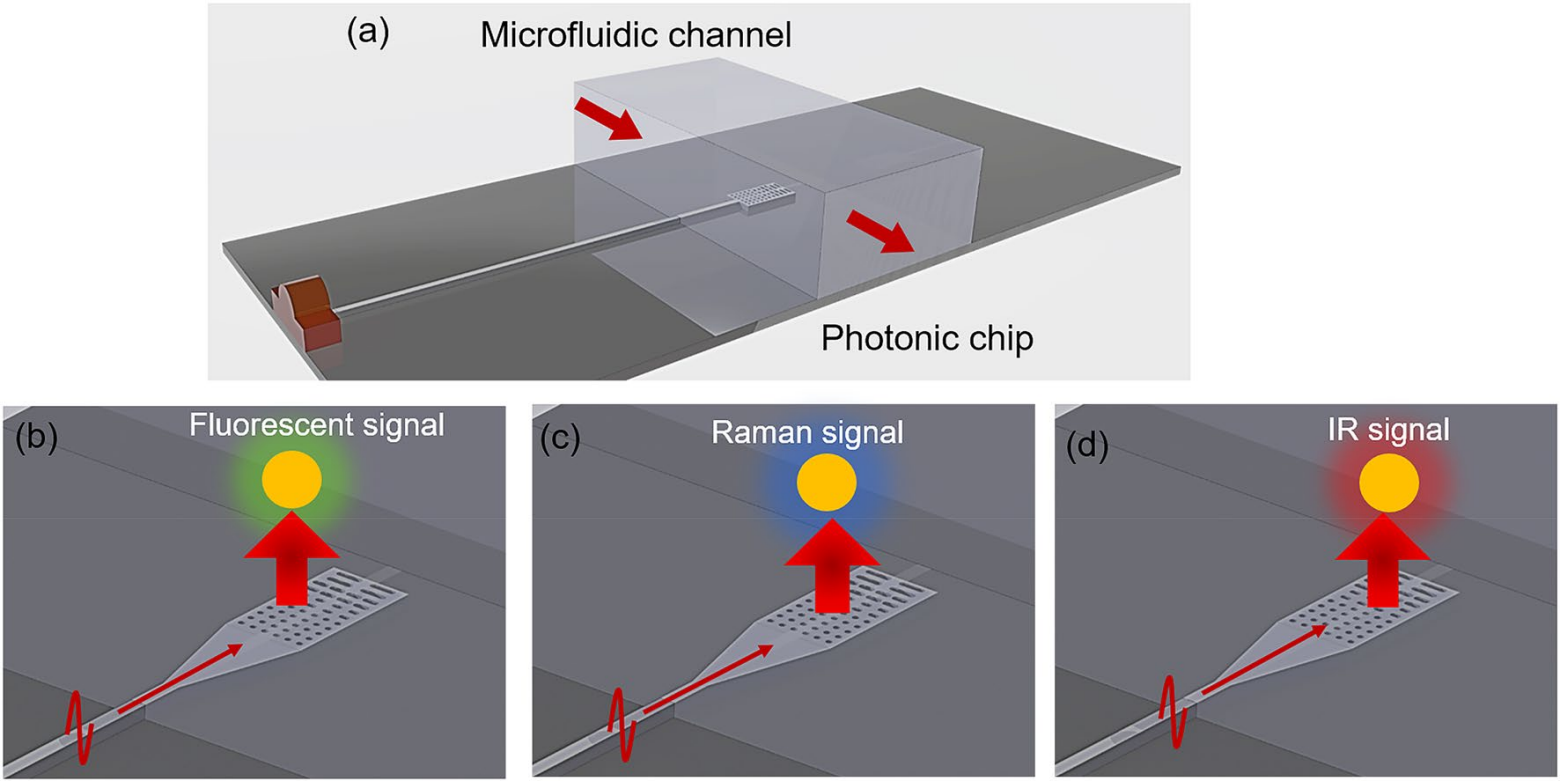
Opto-fluidic Lab on the chip sensing system. (**a**) Photonic waveguide technology integrated with a microfluidic chip to perform high throughput detection and sensing. (**b**) Planar photonic waveguide based light excitation and detection for fluorescence spectroscopy. The excitation of the sample (shown in red) generates a fluorescent signal (shown in green) detected by adjoining detectors (**c**) Planar waveguide-based light excitation for on-chip Raman spectroscopy. The Raman signals are represented in blue. (**d**) Planar waveguide-based light excitation for on-chip IR spectroscopy where excitation and detection signals are shown in red. Figure created in Solidworks Standard.

In this work, we use an inverse modeling approach to obtain an optimally designed metasurface photonic grating structure. The design target for the structure is to create a wide collimated beam, which is useful for on-chip optical excitation and detection^24, 25^ in bio-sensing applications. Figure 1 shows a schematic of the associated integration of the meta surface with varieties of opto-fluidic sensors. We present finite difference time domain (FDTD) modeling, design optimization, device fabrication and experimental demonstration of a meta collimator; we compare beam characteristics with that from conventional grating designs.

## Results

### Meta collimator design and modeling

**Figure 2:**
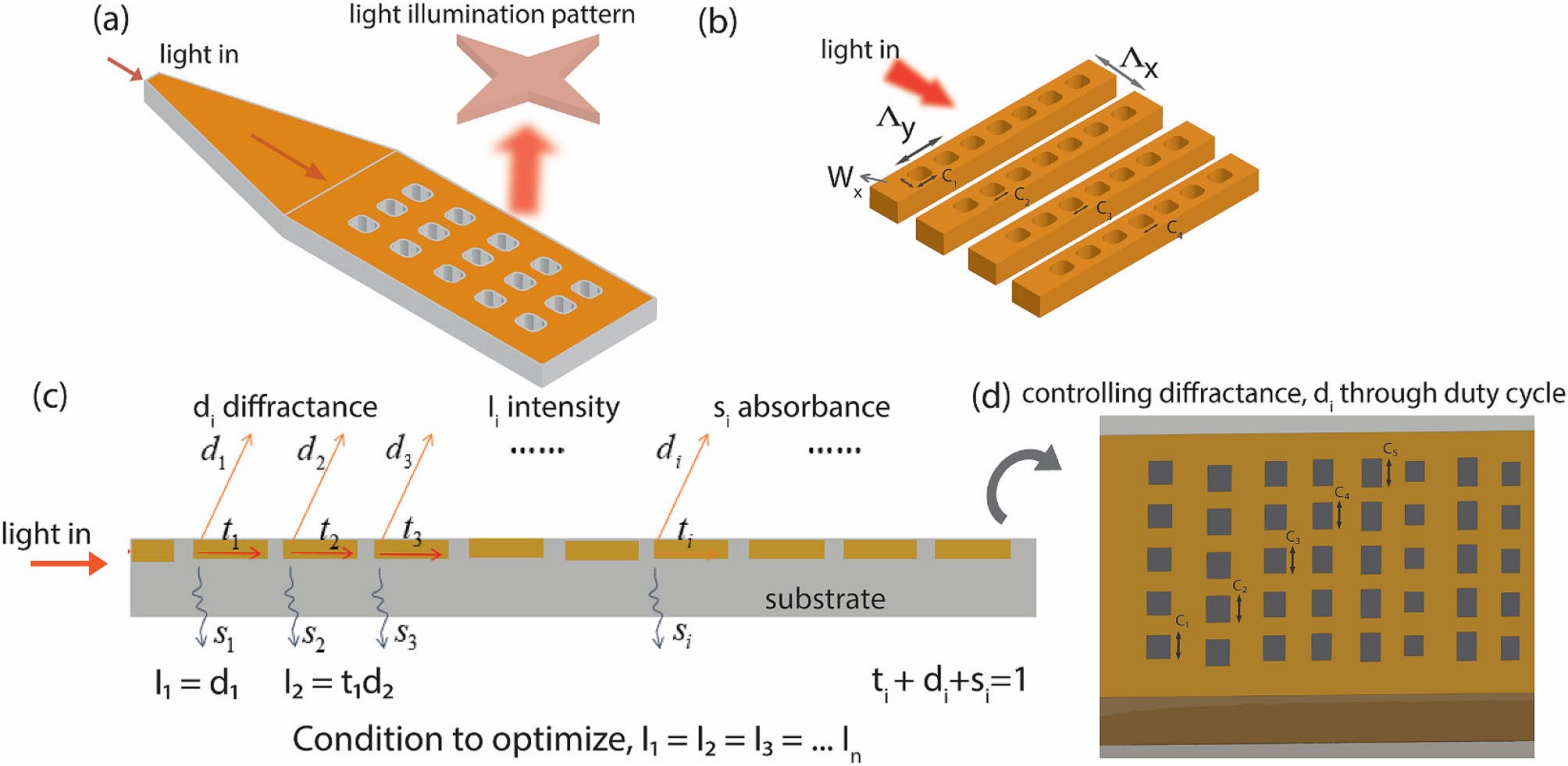
(**a**) Meta grating structure with rectangular scatterers in each grating row. (**b**) The rectangular scatterers are dispersed within a row and their characteristics are determined by the duty cycle (C) and their period ($\Lambda_{x}$, $\Lambda_{y}$). Each row has a different characteristic of rectangular scatterers that determine how much they diffract the light off the plane to illuminate the sample. (**c**) Light propagation in the metasurface is easily understood through an effective mirror model where the light gets diffraction (**d**), absorbed (s) and transmitted (t) through the individual row. Figure created in Adobe Illustrator.

Conventional waveguide-based gratings are composed of multiple identical diffracting grooves along the direction of light propagation; the grooves diffract light out-of-the-plane at a specified angle (Fig. 2a). The power of the emitted light decreases exponentially as it progresses along the waveguide^17, 26^. In order to achieve uniform emission along the waveguide, the amount of energy diffracted from each diffracting groove must be set in proportion to the power loss along the optical path. The power loss is controlled by designing a unique arrangement of light-diffractors along the direction of light propagation. To optimize emission, we mathematically relate critical design parameters, as defined in the schematic shown in Fig. 2b, to light diffraction properties of a meta surface. We define rectangular light scattering grooves in the grating structure by their duty cycle (C), row period ($\Lambda_{y}$) along the transverse direction, and line period ($\Lambda_{x}$) along the propagation direction, as shown in Fig. 2b.

The photonic waveguide terminating with the meta surface offers a high index region to support orthogonally polarized modes propagating along the axial direction. Assuming the incoming optical wave in the waveguide is of the form $E_{0}^{inc} (y,z)e^{i(\beta x - \omega t)}$, where $E_{0}^{inc} (y,z)$ is the amplitude of the electric field and $\beta$ is the propagation constant, the diffracted beam profile is given by, $E_{0}^{diff} (y,z)e^{{i(k_{xn} x - \omega t)}}$ with the propagation constant as

1 $$k_{{xn}} = \beta _{n} + i\alpha = \beta _{0} + \frac{{2n\pi }}{{\Lambda _{x} }} + i\alpha$$

Here, $\beta_{n}$ is the propagation constant of the diffracted beam that depends on the periodicity of meta scatterers in the x direction, $\alpha$ is the energy leakage factor and *n* refers to the diffraction order. The angle of diffraction measured from the vertical axis, for nth order of diffraction is given by

2 $$\phi_{n} = \sin^{ - 1} \left( {\frac{{\beta_{n} }}{{k_{0} }}} \right).$$

Specifically, for off-the-plane diffraction of the beam with no higher order diffraction patterns, it is customary to satisfy the following conditions, as shown elsewhere^26^,

3 $$\left| {n_{wg} - \left( {\frac{\lambda }{{\Lambda_{x} }}} \right)} \right| \le \sqrt {\varepsilon_{a} } = 1,\;\;\;2\left( {\frac{\lambda }{{\Lambda_{x} }}} \right) - n_{wg} > \sqrt {\varepsilon_{{SiO_{2} }} }$$

where $n_{wg}$ is the effective index of the waveguide and $\varepsilon_{a}$ is the permittivity of the cladding (we have assumed it to be air). In case of an opto-fluidic integrated sensor, we replace permittivity of air with that of $\varepsilon$ in surrounding liquid.

Further, the leakage energy in the diffracted beam is given by^26, 27^,

4 $$\alpha = \alpha_{h} (\omega ,D,ff)(\varepsilon_{wg} - \varepsilon_{a} )^{2} \sin^{2} (\pi \times ff)$$

Here, $\alpha_{h} (\omega ,D,ff)$ is the coefficient that is a strong function of light wave frequency ($\omega$), etch depth (D) and fill factor (ff) of the meta scatterers. $\varepsilon_{wg}$ and $\varepsilon_{a}$ are the permittivity of the waveguide and air respectively. Therefore, the critical parameters to control light diffraction are row and line period (in y and x direction respectively) and duty cycles of the scatterers (that controls the width of scatterers). Line period ($\Lambda_{x}$) controls the angle of diffraction, $\phi_{1}$ and line period ($\Lambda_{y}$) along with the duty cycles modulates the effective permittivity of the individual row (explained by the effective permittivity theory).

As a demonstration, we maintain the width, *w*, and length, *l* of the metasurface as 10 μm and 20 μm respectively (Fig. 2a). The center wavelength of the emission spectrum is set to be in the C-band (1550 nm). Further, we ensure that the device can operate over a wide (~ 70 nm) range of wavelengths while optimizing its structure for uniformity.

In our methodology, the optimization of the meta-grating is divided into two stages. The first optimization stage initializes the duty cycle of an individual row in the meta structure and then performs an iterative gradient descent inverse optimization to collimate the output beam. Gradient descent has many advantages, such as enabling the optimization of the photonic structure with relatively large degrees of freedom as compared to other gradient-free optimization schemes. Further, it requires fewer simulation steps and does not rely on parametric extensive search or random mathematical perturbations to find optimum values.

### Parameter initialization

As the gradient descent method is highly sensitive to initial conditions, it is a good practice to initialize the parameters with relevant values. To find the initial values, we use an *effective mirror model* for the meta grating structure. The grating grooves can be approximated by the *cascaded mirror model* to understand light propagation through the structure^28^. Figure 2c shows the meta grating structure with its cascade mirror model. Transmission, diffraction, and scattering coefficients of the diffracting groove are assumed as *t*, *d*, and *s,* respectively. Diffraction intensity output from the first diffracting groove is proportional to *d*, and is given by,

5 $$I_{1} = d_{1}$$

Using the cascaded mirror model, we can write intensity *I*_*2*_ and *I*_*n*_ in general, given as,

6 $$I_{2} = t_{1} d_{2}$$

7 $$I_{n} = t_{1} t_{2} \ldots t_{n - 1} d_{n}$$

The collimated beam requires uniform emission from an individual groove. Mathematically, the condition is represented as,

8 $$I_{1} = I_{2} = \cdots = I_{n} = \cdots$$

Substituting the equations (1), (2), (3) in the condition (4) results in

9 $$d_{1} = t_{1} d_{2} = t_{1} t_{2} d_{3} = \cdots = t_{1} t_{2} \ldots t_{n - 1} d_{n}$$

**Figure 3:**
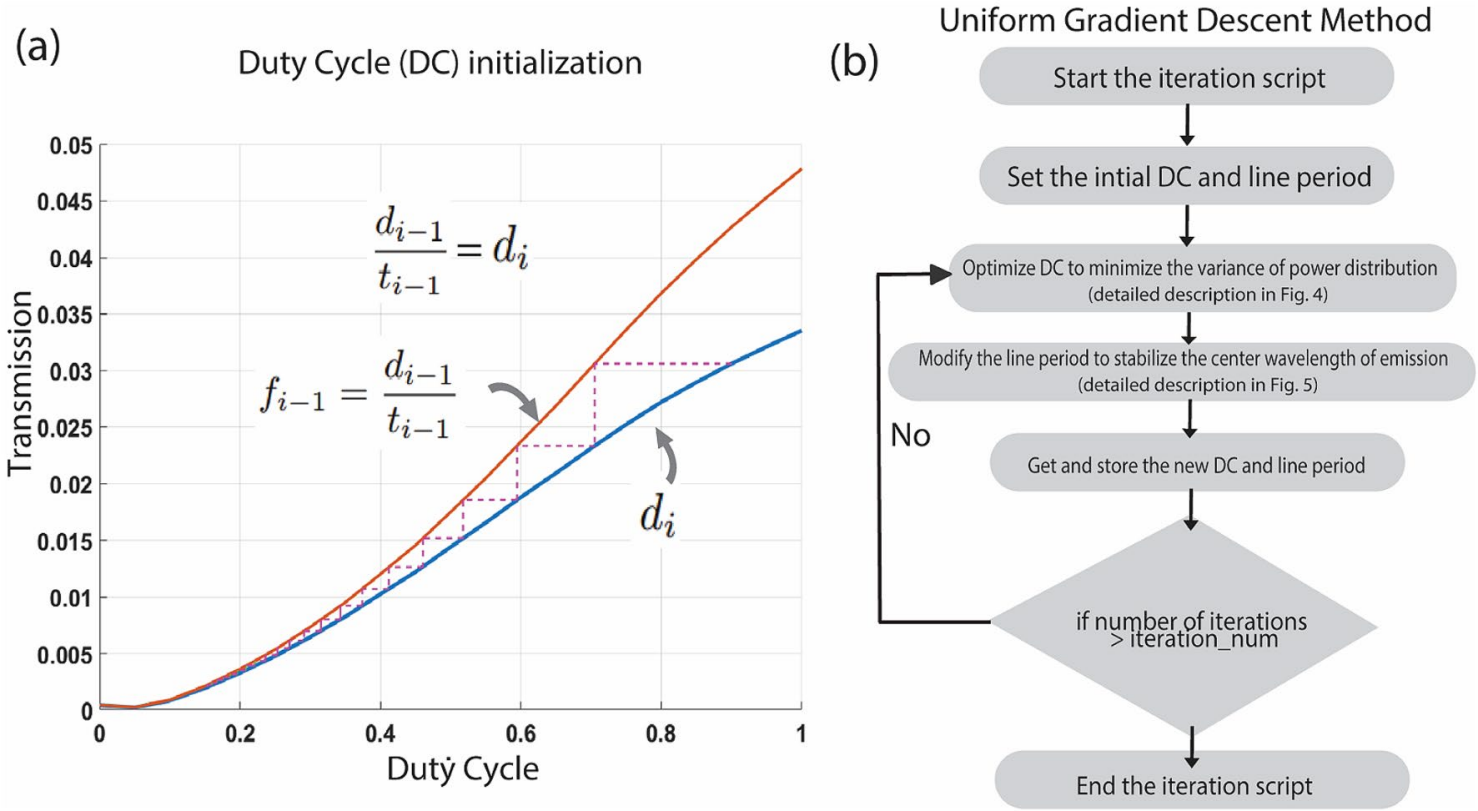
This is the control flow chart of the iteration algorithm. We first set the initial duty cycle and row period, then use gradient descent method to tune the duty cycle and optimize the variance of power distribution. This may cause a shift in central wavelength; thus, we modify the groove period to stabilize it. We obtain a duty cycle and groove period in every loop and iterate until termination constraints are met.

Assuming $f_{i} (x)$, $d_{i} (x)$ as functions of the duty cycle, *x* and width *w,* of the groove. For constant width, we evaluate over a range of values for the duty cycle and obtain the functions, $f_{i} (x)$ and $d_{i} (x)$. Figure 3a shows both the functions plotted for different values of the duty cycle, *x*.

Using the plot of the functions, we initialize the duty cycle for individual meta surface row to satisfy the condition given by,

10 $$f_{i - 1} = \frac{{d_{i - 1} }}{{t_{i - 1} }} = d_{i}$$

### Iteration method

After the initialization process as explained above, we optimize the duty cycle of the individual grating groove in the meta-surface and obtain a collimated beam output. Lumerical FDTD software is used to physically model the diffracted power. Individual row power output increases with an increase in its duty cycle. We calculate the spatial distribution of diffracted power and its variance, then tune the duty cycle of each groove to minimize the variance across the meta scatterers rows (Fig. 2d). Every update in the iterative algorithm is performed as

11 $$C_{i} = C_{i} - \delta \frac{{P_{i} - P_{average} }}{{P_{average} }} = C_{i} - \delta \frac{{I_{i} A_{i} - I_{average} A_{total} }}{{I_{average} A}}$$

where *i* is the row number, $\delta$ is the learning rate of the *gradient descent* method and $P_{i}$ is the output power calculated by integrating the intensity over the *i*th row area of meta surface and $P_{average}$ is the average power over the entire meta surface.

**Figure 4:**
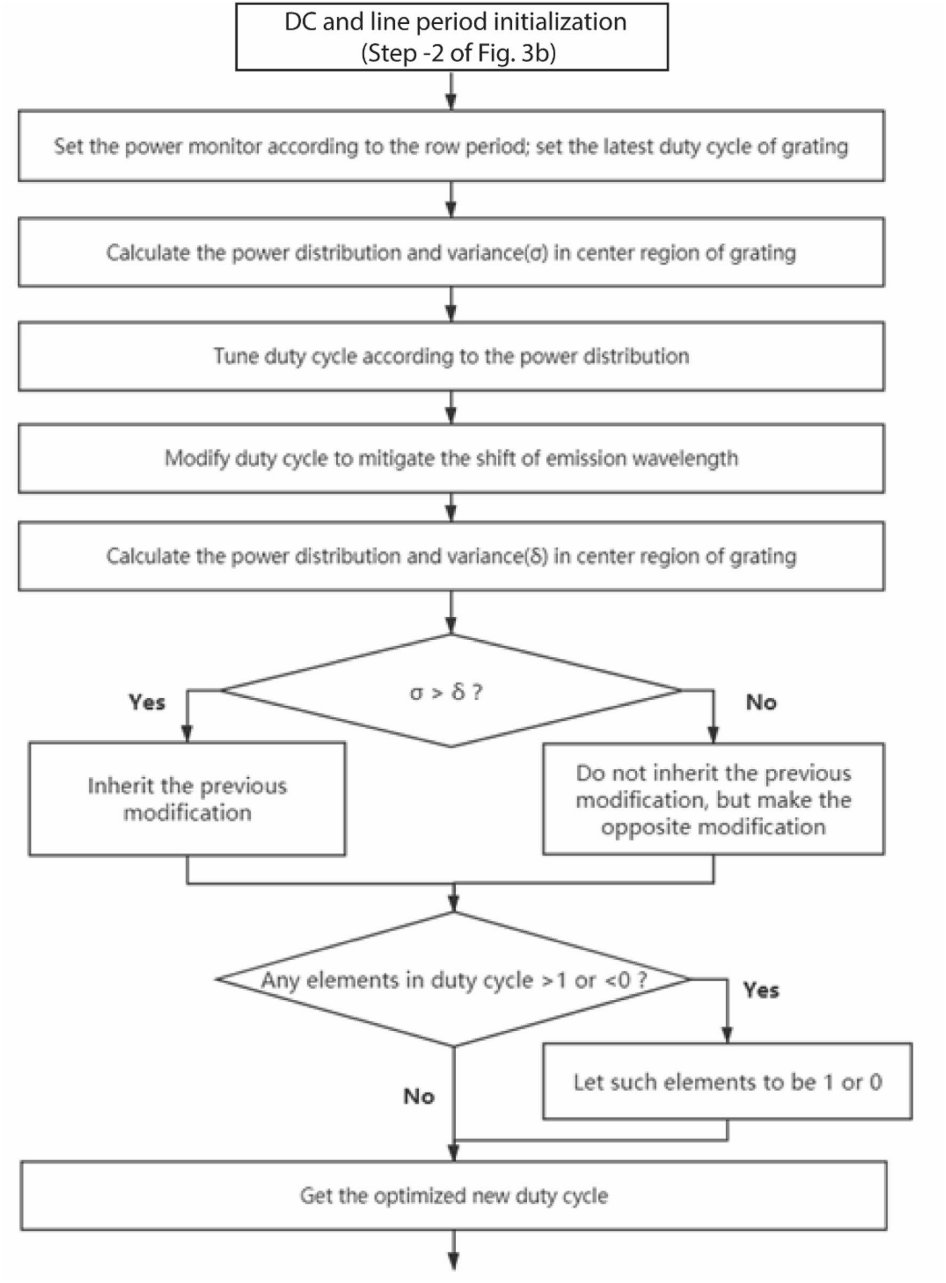
Detailed control flow chart of “Optimize duty cycle (C) to minimize the variance of power distribution” in Fig. 3b.

**Figure 5:**
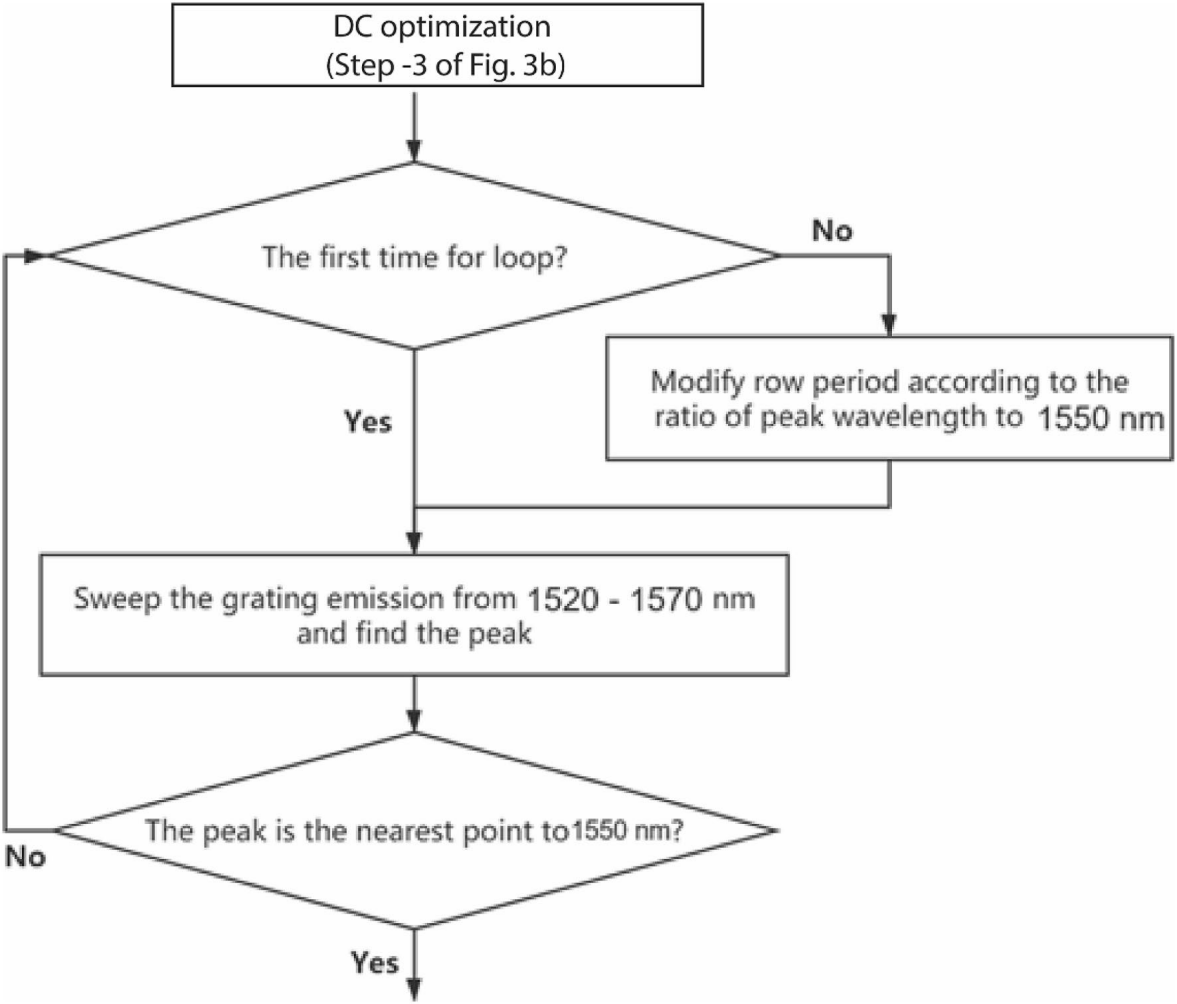
Detailed control flow chart of the “Modify groove period to stabilize the center wavelength of emission” in Fig. 3b.

Figure 3b shows the control flow chart of the optimization routine. With every iteration, the uniformity in the power distribution of the beam collimation increases. When the duty cycle and the grating period change, the effective index of the structure changes, as a result of which the center wavelength of grating emission also shifts. This may result in poor convergence of the duty cycle in the iterative procedure. Therefore, the central emission wavelength is stabilized by modifying the grating period. We add an additional step in the algorithm to tune the line period ($\Lambda_{x}$) and stabilize the emission wavelength. Some spatial randomness is incorporated in each row to avoid the lattice diffraction patterns in the profile. Figures 4 and 5 explain the process where in the duty cycle and row period optimization are visualized respectively, in the flow chart. The method is wavelength independent and can be used across different photonic materials as well. Here, we present the SiN based photonic design operating at C and L band, the most commonly used wavelength ranges in communication industries.

## Discussion

**Figure 6:**
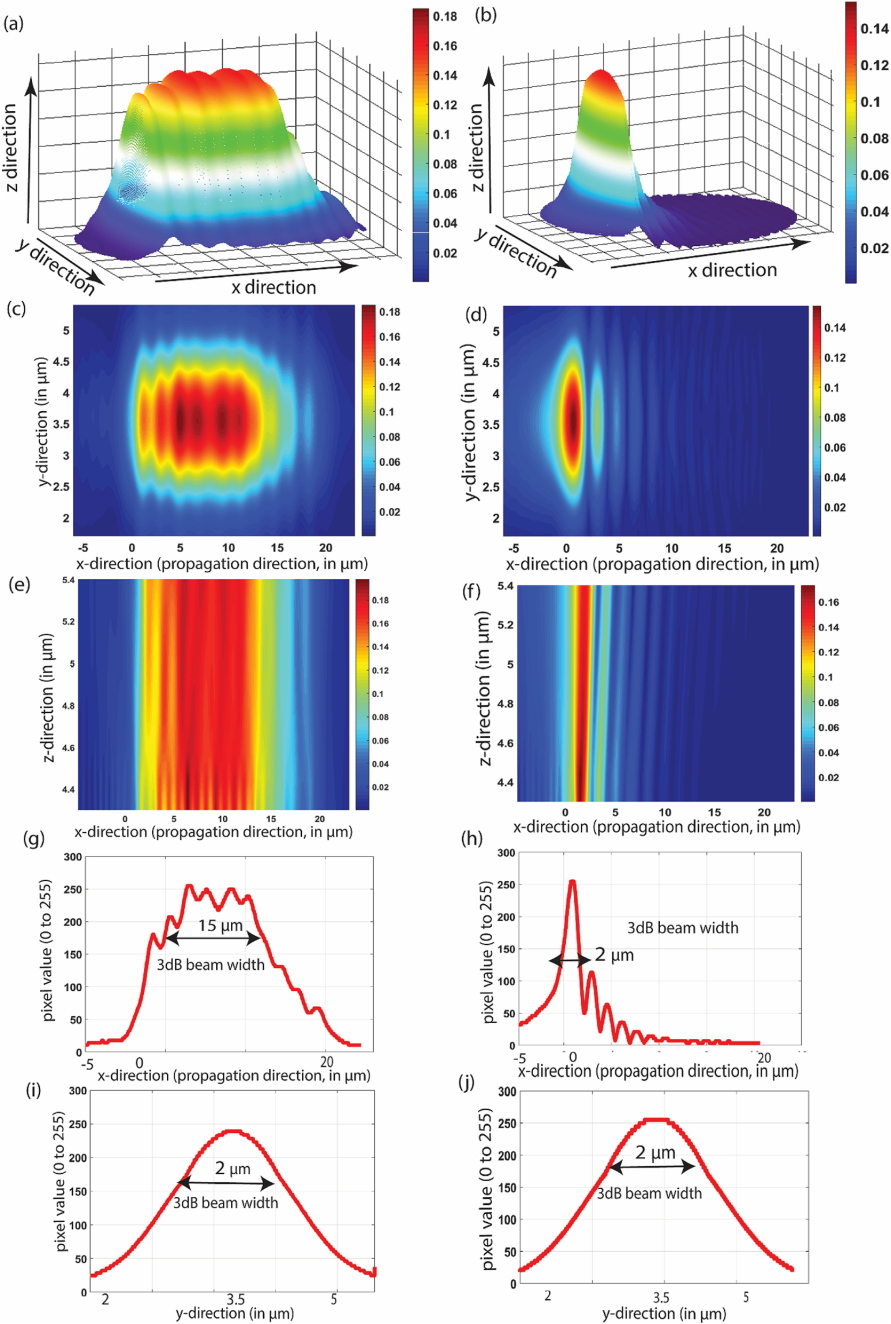
Diffraction beam profile from the meta surface and the conventional binary grating structure. (**a**,**b**) The 3D beam profiles. (**c**,**d**) The top view of the beam profile from the meta surface and conventional gratings respectively. (**e**,**f**) Side view of the beam profile from the metasurface and conventional gratings respectively. (**g**,**h**) The 3 dB beam width analysis in the x direction for the metasurface and conventional gratings respectively. (**i**,**j**) The 3 dB beam width analysis in the y direction for the metasurface and conventional gratings respectively. Figure created in MATLAB2018b.

Results obtained from the optimization process are shown in Fig. 6. It shows 3D, top and side view of the output beam from the optimized metasurface and compares it with a conventional binary grating structure. We perform a quantitative beam analysis to compare the 3 dB beam spot and uniformity in the illumination pattern as shown in Fig. 6. We conclude that optimization improves the beam spot by 8 times in the direction of propagation, assuming a constant surface area (20 $\times$ 5 µm^2^). After optimization, the result is a collimated beam with approximately ~ 50 nm of operating bandwidth.

### Design performance and characterization

**Figure 7:**
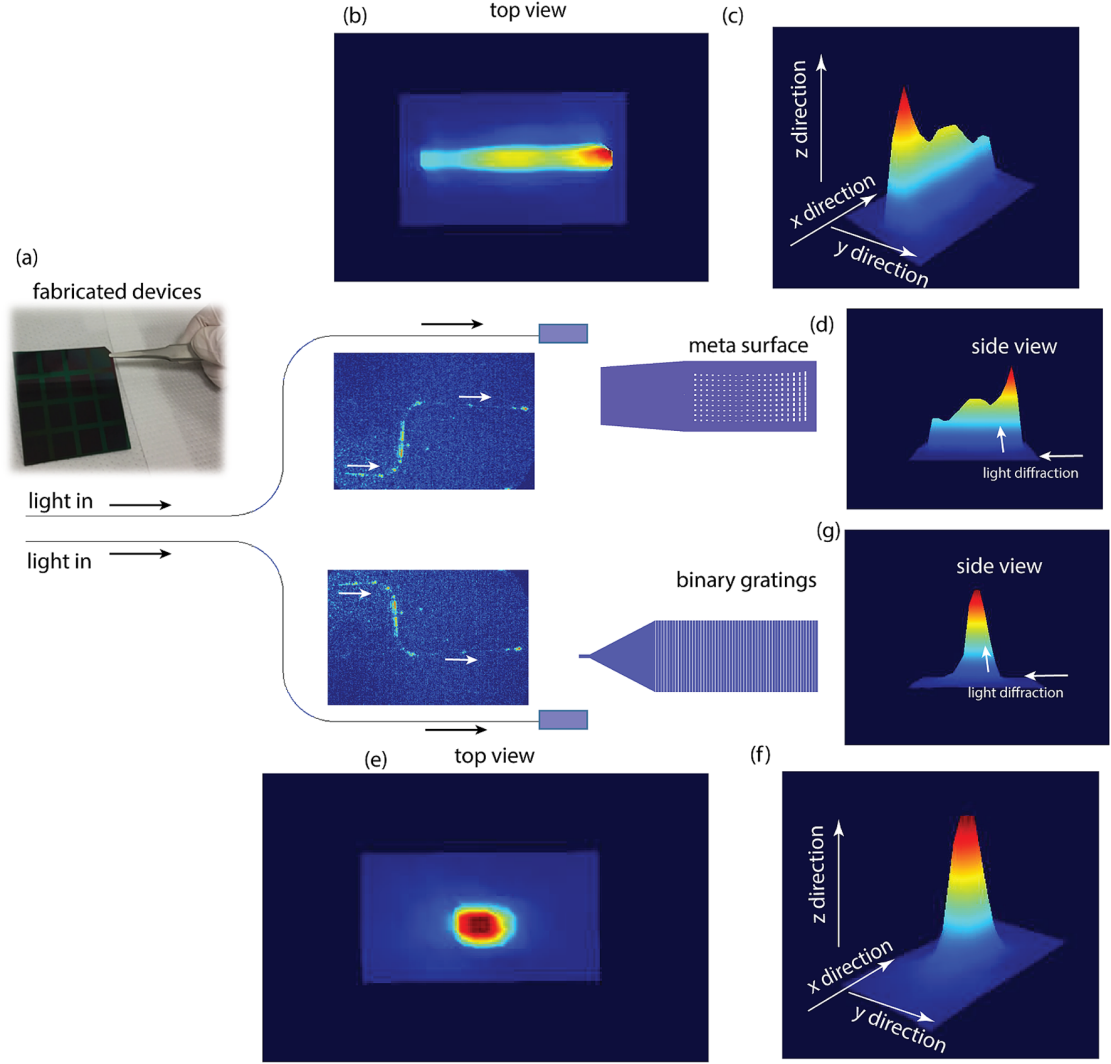
Experimental comparison of metasurface and conventional binary grating-based diffractor. (**a**) The fabricated sample. (**b**,**e**) Microscopic top view of the light diffraction from the photonic meta surface and binary gratings. (**c**,**d**,**f**,**g**) 3D and side view of the beam profiles from the metasurface and the binary grating structure. Figure created in CinCam beam profiler.

We compare the fabricated photonic meta-surface against a uniform binary grating-based diffractor. Figure 7 shows the experimental analysis of the beam profile for the micro-structures. We show a 10× magnified version of the beam profile from conventional gratings for better visualization against the beam profile from the meta structure. Figure 7a shows the microscopic view of the light propagating in the photonic waveguides. Figure 7d,g compare the side view of the beam pattern. The respective top views are shown in Fig. 7b,e. We find that the power delivered through the meta-surface is approximately 5 times that of the conventional grating surface. The delivered power increased from about 4 P.U. to 27 P.U. The 3 dB width of the beam spot increased from 100 μm to  ~ 300 μm with the meta-surface (for footprint of 200 × 50 μm^2^) enabling greater light illumination efficiency of the on-chip device.

### Outlook

We demonstrate a meta-surface based photonic beam collimator for developing chip-scale spectroscopic methods such as in on-chip optofluidic probing. The beam collimator is an attractive option for miniaturized excitation sources. We adopt an inverse design strategy to optimize the meta structure. The optimization was based on a gradient descent method where the parameters were initialized using an effective mirror model. The designed meta-surface is successfully fabricated and experimentally characterized. We compare its beam profiling performance with a conventional binary grating structure. Such an excitation source when integrated with a planar waveguide-based photonic detector/photo detector has the potential to miniaturize optical spectroscopy.

## Materials and methods

### Device fabrication

**Figure 8:**
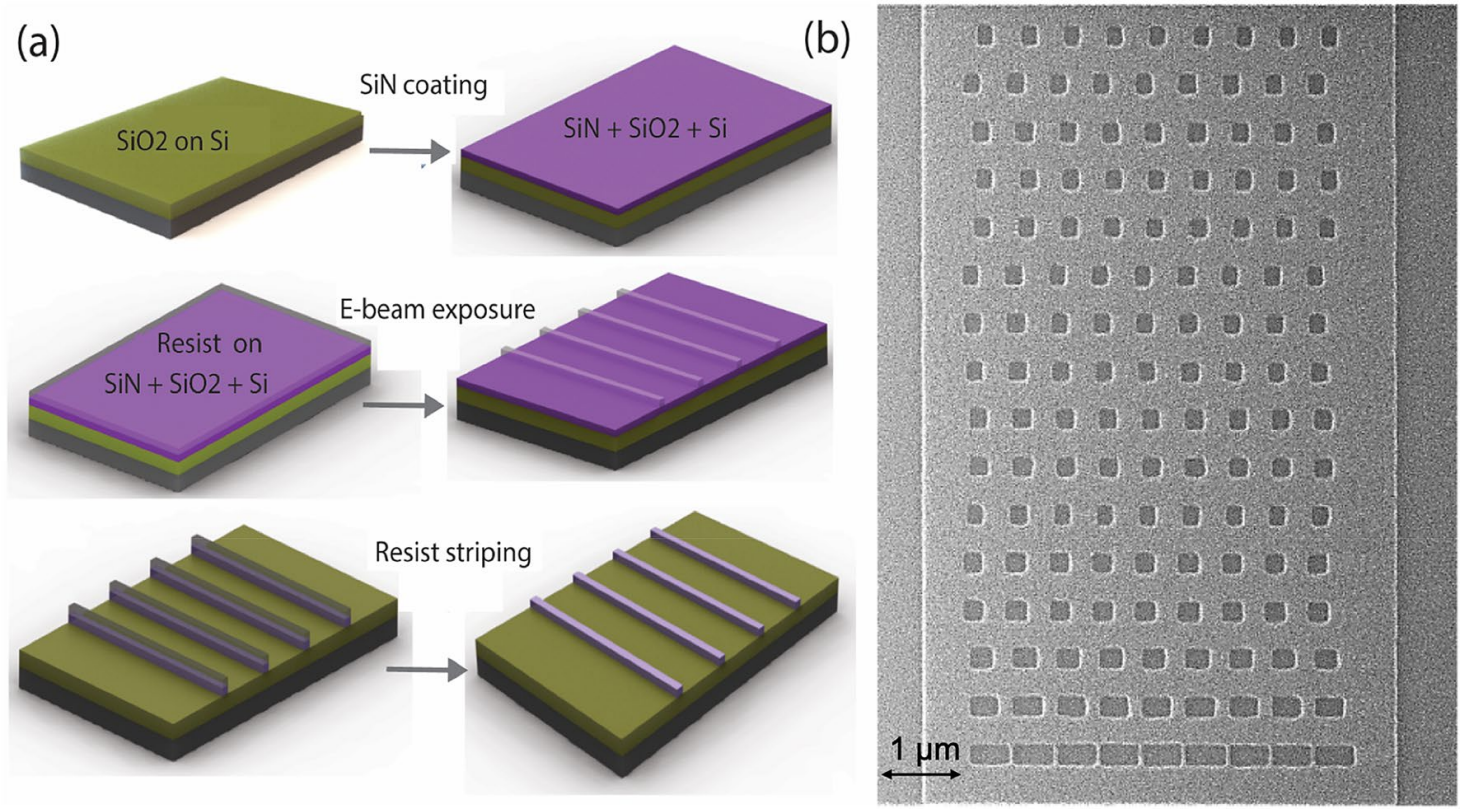
Process flow steps involved in the fabrication of the metasurface (created in Solidworks Standard). (**b**) He ion microscopy of the patterned structure on top of Silicon nitride.

The device fabrication involves a single layer of patterning on Si_3_N_4_ based photonics, as shown in Fig. 8. A low-pressure chemical vapor deposition (LPCVD) system is used to deposit 400 nm thick silicon nitride layer on a 6-in. silicon dioxide wafer (3-micron oxide on Si substrate). The gratings and waveguides are patterned on silicon nitride on-insulator substrate via e-beam lithography and followed by reactive ion etching to define the geometry of the grating structures. Fluorine chemistry with a gas mixture of CH_4_ and CHF_3_ is used in the dry etching step. The leftover resist is stripped using oxygen cleaning and acetone rinsing. The outlined e-beam based fabrication process enables us to obtain critical dimensions as low as ~ 70 nm there by reducing the fabrication tolerances to about ~ 12–14%.

### Experimental characterization setup

**Figure 9:**
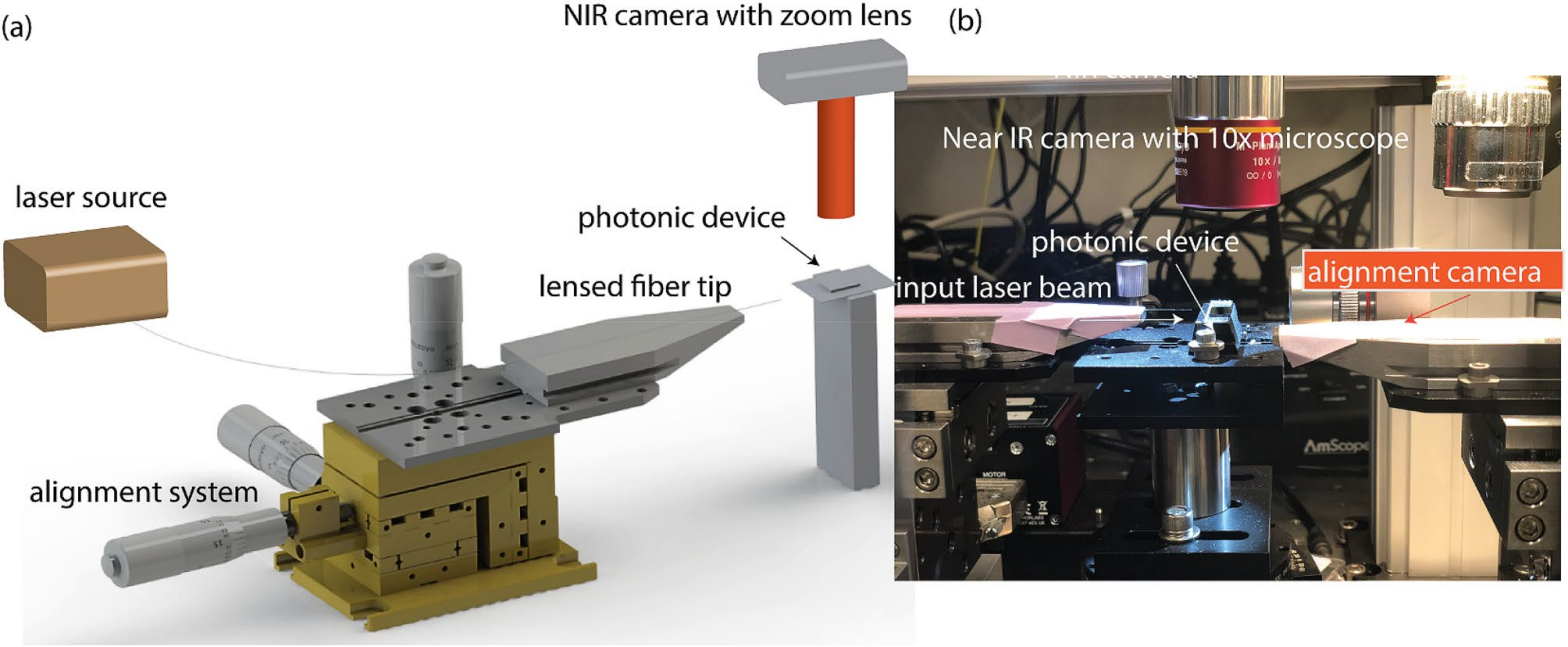
The experimental characterization workbench setup for the photonic device characterization. (**a**) Schematic of the setup (created in Solidworks Standard). (**b**) Experimental setup with NIR and visible microscopic cameras to monitor the device performance.

Figure 9 shows the experimental setup used to characterize the photonic device. We use an Agilent 81640A (tunable across C and L band) laser system coupled to an 8164A optical test mainframe. The output of the laser is fiber-coupled terminating with a lensed tip. The lensed tip fibers (with 1 μm working distance) are procured from Nanonics Imaging Ltd (Jerusalem, Israel). The lensed tip fiber is aligned to the photonic device through 3-axis stage (XYZ Linear Stage, ULTRAlign) obtained from Newport Inc. We use a NIR camera from microViewer Inc to align the fiber tip to the waveguide. During the experimental characterization, the CinCam InGasAS SWI camera (Axiom Optics, Somerville, MA) is mounted upside down to analyze the beam profile.

## Data Availability

Lumerical script files and any other accompanied codes used for modeling of the meta-surface are available from the corresponding authors upon reasonable request.
